# C-Reactive Protein as a Diagnostic Marker in Dogs: A Review

**DOI:** 10.3390/ani12202888

**Published:** 2022-10-21

**Authors:** Katarzyna Malin, Olga Witkowska-Piłaszewicz

**Affiliations:** Department of Large Animals Diseases and Clinic, Institute of Veterinary Medicine, Warsaw University of Life Sciences, 02-787 Warsaw, Poland

**Keywords:** C-reactive protein, CRP, marker, diagnostic, dog

## Abstract

**Simple Summary:**

C-reactive protein is a major positive acute phase protein in dogs. It is commonly used as a marker of inflammation that, although nonspecific, is highly sensitive. The high clinical value lies in its rapid response and relatively short half-life time; these qualities make the C-reactive protein a good therapeutic guide; among others, it can be used to determine when an antimicrobial therapy could be ceased. Various tests are available on the market and the measurement is becoming a part of routine biochemistry blood panels in many countries. Although it is very useful, especially in conjunction with white blood cell count or other acute phase response proteins measurements, it does not allow a complete evaluation as a single parameter.

**Abstract:**

Acute phase response is a nonspecific reaction to disturbances in homeostasis during which the production of some Acute Phase Proteins (APPs) is stimulated; they are sensitive but nonspecific markers of systemic inflammatory processes. The major positive APP in dogs is the C-reactive protein (CRP). The dynamic of its concentration changes fast, rising and decreasing rapidly with the onset and removal of the inflammatory stimulus. It increases within the first 4–24 h after the stimulus and reaches up to a 50–100-fold increase of the baseline level. It has been documented that this APP’s concentration is elevated during several diseases, such as pyometra, panniculitis, acute pancreatitis, polyarthritis, sepsis, immune-mediated hemolytic anemia, and neoplasia in dogs. In clinical practice, canine CRP is mostly measured to detect and monitor systemic inflammatory activity and the efficacy of treatments, because it is a more sensitive marker than shifts in leukocyte counts. Blood serum CRP concentration is becoming a part of routine biochemistry panels in many countries. In this article, changes in CRP concentration and its clinical application in healthy and diseased dogs are discussed.

## 1. Introduction

**Figure 1:**
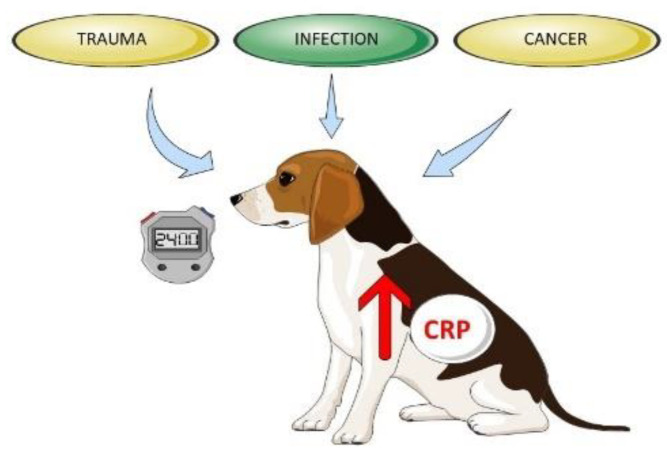
Factors inducing acute phase response, including CRP.

Biomarkers are widely discussed in veterinary medicine, mostly those that can be easily measured in blood. However, finding the most sensitive and specific one is often impossible, especially in inflammatory disorders. The acute phase response (APR) is the initial nonspecific systemic reaction induced by disturbances of homeostasis such as injuries, infection, neoplasia, and other pathologies (Figure 1). After such stimulation, the release of acute phase proteins (APPs, mostly from the liver to the bloodstream) occurs. Thus, the evaluation of their blood concentrations changes seems to be a good, real-time indicator of ongoing systemic inflammation. 

APPs are proteins whose concentration changes a minimum 25% [1,2] in response to inflammatory cytokines (e.g., IL-1, IL-6, TNFα); they are considered positive or negative, depending on whether the concentration rises or decreases, respectively. In addition, based on their magnitude, they are classified as major (APPs that increase 10–1000-fold, reaching a peak 24–48 h after the insult), moderate (5–10-fold, peaking around 48–72 h after insult), and minor (less than 2-fold increase). APPs share several anti-inflammatory and immunomodulatory functions such as the promotion of phagocytosis, induction of cytokine production, inhibition of chemotaxis, and modulation of neutrophil function, among others [1,2]. 

**Figure 2:**
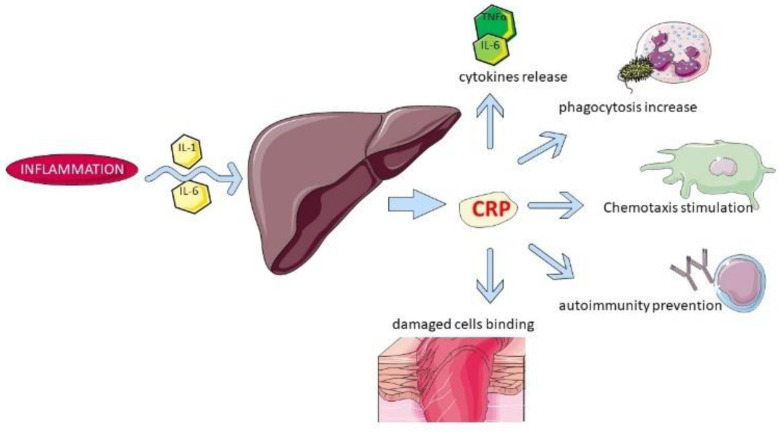
Some of the biological functions of CRP.

The changes in APPs are species-specific. C-reactive protein (CRP) is a major positive APP in dogs, and it is the most sensitive APP in this species [3]. Canine CRP has a molecular weight of approximately 100 kDa and consists of 5 subunits with an apparent molecular weight (MW) of approximately 20 kDa each [4]. It is mostly produced after proinflammatory stimulation by cytokines such as IL-1, IL-6, and tumor necrosis factor-α (TNF-α) [5]. The blood concentration of CRP changes dramatically within 4–6 h after the inflammatory stimulus and reaches its maximum concentration after about 24–48 h, which in most cases reflects the physical degree of tissue trauma. Some of the CRP’s biological functions are shown in Figure 2.

An increase in CRP should not be considered a marker of etiologic diagnosis due to its low specificity. However, it is highly sensitive and has great diagnostic value in the presence of subclinical disease. CRP’s increase in several inflammatory or non-inflammatory conditions [6,7,8,9,10,11,12] is shown in Table 1. Thus, such an assay should be taken into consideration when deciding on early treatment and can guide the recovery. 

## 2. Clinical Resources Available in Veterinary Medicine

There are various methods to measure CRP, including ELISA, immunoturbidometric assays, latex agglutination, and a time-resolved immunofluorometric assay [5]. Only validated assays should be used for the measurement of all APPs because of the cross-reactivity of the used antibodies and different limits of detection (LOD), especially when different body fluids are evaluated (ex. blood vs. synovial fluid) [26]. Assays available in veterinary medicine are summarized in Table 2.

The immunoturbidimetric assay is the most commonly used in clinical laboratories when rapid results are needed to measure the CRP blood level in dogs. Due to its higher precision compared to ELISA assays, it is also commonly used for research projects. The samples can be stored for later analysis, as CRP is resistant to −10 °C for up to 4 months of storage [39] and even longer in lower temperatures [34].

In most clinical veterinary laboratories, only blood serum is recommended for routine CRP analysis. Various anticoagulants can also be used, but they may distort the result of the measurement. In one study, values for CRP were significantly lower in samples with trisodium citrate than with serum (30%), whereas there were no differences when lithium heparin or tripotassium ethylenediamine tetraacetic acid (EDTA K3) were used [40]. EDTA and citrate both remove calcium to prevent coagulation while heparin acts instead by binding to antithrombin III, which suggests that these mechanisms are not responsible for the observed disturbances and so far, the reason is unknown. However, the levels of CRP in samples used for this anticoagulant study were very low; thus, it should be interpreted with caution. On the other hand, human studies showed no difference in CRP serum and plasma concentration) [41]. 

According to several studies various values are considered the upper point value of the reference interval (RI): <5 μg/mL [1,42], <10 μg/mL [43], 0.22–4.04 μg/mL [44], 0.8–16.4 μg/mL [45], 8.4 ± 4.6 μg/mL [37], 0.48 ± 0.17 μg/mL [46]. Therefore, RIs reported in the literature are variable, however, the reported ranges are not extremely wide (the URLs vary between approx. 5 and approx. 20 μg/mL). The possible sources of this variation among studies or laboratories are based on the method, protocol, reagent and anticoagulant, and instruments storage, as previously discussed [9]. The reading may also be affected by lipemia, hemolysis, and bilirubinemia when ELISA is used, but it is not observed in immunoturbidimetric assays [2,29]. Especially, the measurement may vary to the clinically critical extent when polyclonal human antibodies are used, and the machinery is calibrated with human CRP. Such practice requires batch-to-batch validation since different cross-reactivity can be expected in various batches [47].

Although highly accurate, species-specific validated assays are available worldwide, CRP should never be interpreted as a single parameter. Some laboratories include CRP as a part of biochemistry panels, but the Seven Points Plan, published in 2008 and updated in 2019 [48], recommends a broader APPs profile that includes major positive, major negative, and moderate acute phase proteins to be implemented because of the high diagnostic value of their divergence. As an example, the authors explain that a simultaneous increase in haptoglobin, accompanied by a normal level of CRP, could suggest a possible diagnosis of hyperadrenocorticism. Another major part of an evaluation of an inflammatory process is the white blood cell count (WBC); but APPs are more stable than WBC [48]. WBC shifts even when inflammation is not involved. Neutrophilia and lymphopenia can be a result of the inhibition of margination, and it is well known that stress and glucocorticoid therapy can induce both [49]. When in doubt, even the measurement of CRP alone can help determine the underlying cause of such shifts.

## 3. CRP Concentrations in Healthy Dogs

Compared with other markers, small changes in CRP over time may be relevant for diagnostic or prognostic purposes. It is best recommended to obtain baseline CRP concentration in a healthy individual to then compare with further results [50]; this is the most adequate approach due to the wide reference values and individual differences. However, it is rarely done in veterinary medicine. In clinical practice, baseline CRP is a part of routine biochemistry panels in many countries, but it is not the worldwide standard yet.

In countries where it is not included, it should be recommended to run it in addition; in cases where the value was not additionally requested to be recorded before the potential sickness, the most practical recommendation is that the patient’s health status can still be monitored through repeated measurements of CRP over time during the follow-ups, to assess possible changes compared with the initial value (which also should be compared to the population-based RI).

There may exist some breed-related differences. It was documented that in clinically healthy Miniature Schnauzers (MS) [51] (median 4.0 μg/mL, minimum–maximum 0–18.2 μg/mL), CRP was found to be higher than in non-MS of the study (median 0.1 μg/mL, minimum–maximum 0–10.7 μg/mL). It is unclear whether the baseline serum CRP concentration could predict or be correlated with hyperlipidemia and pancreatitis in MS dogs, to which they are prone, but an association between hypertriglyceridemia and pancreatitis is well documented in humans [52].

No changes in CRP concentration were observed over day and night circadian rhythms [44]. Shifts are noticeable in pregnant bitches, peaking at a 2–4-fold increase (70–90 μg/mL), 30–45 days after the ovulation [53]. Even though the concentration drops after the peak, it remains elevated until the parturition [54]. So far, no evidence suggests differences in CRP levels as dogs age. However, in one study, where dogs were inoculated with turpentine oil, dogs 1 month of age showed a maximum 15- fold CRP rise, while 3 months and older increased the value up to 26-fold [55]. Therefore, CRP might not be diagnostic in canines younger than 3 months, because the response to inflammation is significantly lower than in those below 3 months of age

## 4. Physical Exercise

Physical activity is associated with a change in APPs concentrations in different species [56] and is connected with duration and type of exercise. In a study focused on the influence of short-duration exercise in racing Greyhounds, a decrease in CRP concentration was found, whereas other APPs levels increased in 2 h after the exercise (e.g., serum amyloid A and haptoglobin) [57]. In German Shepherds, CRP was measured right before and 6 h after the exercise; showing a lower than 1-fold increase (shift from 40.69 ± 16.48 μg/mL to 45.00 ± 13.37 μg/mL) meaning the change was statistically insignificant [58]. Disturbances in glucose homeostasis, acid-base, and electrolyte balance were suggested as the underlying causes of the multi-level reaction of the organism.

Other authors measured CRP concentrations over a shorter period in hunting dogs. With the exercise’s duration of 3 h, the assay was performed immediately before (T0), immediately after (T1), and one hour after it was over (T2) [1]. A significant 4-fold rise was observed (from 1 μg/mL (T0) to 4.5 μg/mL (T1)) in comparison to no changes at T0, T1, and T2 in the control group. Notably, in this study, the CRP value 1 h after the workout has already decreased to the same value as T0. It was discussed by the authors that the overall APPs rise could have to do with subclinical arthritis in these animals, but the white blood cells (WBC) count performed before and after the exercise showed no variability, which likely excludes such a possibility. The CRP increase may be connected with the exercise APR-like response, which was documented in other species [56].

The fact that endurance exercise-induced changes in CRP might be indistinguishable from those caused by an actual inflammatory response was proven by another study [57,58,59]. Sled dogs are elite athletes and can endure days running. Candidates who showed elevated WBC count and/or rectal temperature above 39.4 °C were excluded from the study; 12 healthy, mixed-breed dogs ran 557 km over 4–5 days. Testing 48 h before the workout began and within 20 min of completing it, over a 10-time fold increase was observed (rising from a mean of 22.4 ± 16.3 µg/mL to 263.3 ± 103.8 µg/mL). CRP concentration was also measured in sled dogs completing a 1650-km race and significant changes were observed: CRP (median [range]) start: 18 μg/mL [11,12,13,14,15,16,17,18,19,20,21,22,23,24,25,26,27,28,29,30,31,32,33,34,35,36,37,38,39,40,41,42,43,44,45,46,47,48,49,50,51,52,53,54,55,56,57,58]; midpoint: 119 76 μg/mL [12,13,14,15,16,17,18,19]; finish: 60 μg/mL [12,13,14,15,16,17,60]. In another study, performed on 65 sled dogs, it was documented that CRP concentration increased after a training session and a long-distance race [61]. There, it was confirmed that CRP level was elevated in both finishing and non-finishing dogs. Thus, this APP cannot indicate a dog’s readiness for race. On the other hand, in sled dogs with rhabdomyolysis, CRP level was 2-fold elevated in comparison to the healthy animals (308 vs. 164 μg/mL) [62].

Thus, changes in CRP concentration may be a useful marker for monitoring the physical adaptation in the competing dogs, especially in endurance training. More studies are necessary to establish the patterns of interpretation of the results, however, since the half-life time of CRP is 19 h [63], a clinician should not be surprised by a higher value in an athlete patient, despite the lack of inflammatory disease. Vice versa, whenever an athlete canine requires medical attention midst or shortly after exercise or competition, a dramatic elevation of CRP is to be expected regardless of the emergency.

In conclusion, serum CRP should not be used to determine which animal is capable of physical effort. It is additionally supported by the evidence that degenerative joint diseases do not increase this parameter in the bloodstream [26].

## 5. Bacterial and Viral Etiology Diseases

Bacterial infections can produce the most intense inflammatory processes, and, alongside anaphylaxis, can result in distributive shock resulting in the death of the affected animal [64]. In human medicine, there used to be an outdated generalization that high CRP blood concentration is a marker of somewhat specificity for bacterial infections. Similarly in veterinary medicine, septic pneumonia would be linked to levels above 100 μg/mL in dogs [65]; this approach has shifted in human medicine, and it has been shown that the parameter is rather used to reduce the number of antibiotic treatments prescribed in patients with acute cough [66].

For reference, in a study including 147 dogs with CRP concentrations greater than 100 ug/mL, 86/147 dogs had an associated inflammation, but only 33/147 (22%) of them had a confirmed bacterial infection and 11 dogs had a viral infection. No statistically significant differences were found between infected dogs and any other group (animals with trauma, neoplasia, etc.) [65]. Thus, extremely high CRP concentrations do not allow a simple conclusion of the underlying etiology or identification of bacterial inflammation and cannot be used to excuse the administration of an antibiotic. The authors also concluded that CRP does not rise in an organ-specific manner, nor is it specific for infectious diseases.

In another recent study, such high values of CRP concentration have not been observed. However, a comparison between dogs with sepsis and trauma showed a significantly higher increase in the first group [67]. Additionally, the evaluation of CRP at point-of-admission was a reliable prognostic marker. Similarly in a different publication, although it concluded that sepsis cannot be differentiated from aseptic Systemic Inflammatory Response Syndrome (SIRS) based on CRP, in both cases, a rapid decrease of CRP, rather than initial concentration, was a good prognostic marker [68]. In addition, higher mean CRP concentrations were found in dogs with SIRS or sepsis (165 ± 82 μg/mL) than in those with localized inflammation (108 ± 70 μg/mL) [8].

CRP proves to be useful in guiding antibiotic therapy and allows one to determine when it should be discontinued. An in-depth analysis was run regarding, among other parameters, CRP levels accompanying various respiratory diseases, including the infectious [69]. In a total of 106 dogs, the 22 diagnosed with bacterial pneumonia experienced a significantly higher rise in CRP than dogs with other respiratory diseases (bacterial tracheobronchitis, idiopathic pulmonary fibrosis, chronic bronchitis, cardiogenic pulmonary edema, eosinophilic bronchopneumopathy). In dogs with bacterial pneumonia, the serum CRP was successfully used to guide the duration of antibiotic treatment and it was stopped 5–7 days after the CRP normalization [70]; this finding supports the idea that the APPs evaluation could be used in support of the therapeutic approach (i.e., the need to use the antibiotic or supportive treatment) in veterinary medicine as well to minimalize the risk of creating resistant bacterial strains.

In experimental conditions, where the exact inoculation time is set, more precise diagnostic criteria could be established. CRP concentrations as high as 720 μg/mL (mean 478 μg/mL) were noted 24 h after intratracheal inoculation with *Bordetella bronchiseptica* and later continuously dropped until day 20 [13]. In the dogs that were inoculated again on day 36, the initial peak was not as high. Clinical symptoms, such as coughing, started 1–3 days after the first inoculation, and until day 5, all the dogs showed CRP > 100 μg/mL. In contrast, a comparison of CRP concentrations in dogs with *Bordetella bronchiseptica* infection and aspiration bronchopneumonia was described by other researchers in a retrospective study [10]. In all of the dogs CRP was elevated. However, such dramatic CRP elevation was not recorded at all, in fact, in the aspiration bronchopneumonia group, the increase was significantly higher (20 vs. 118 μg/mL). The findings of both those studies show that patients cannot be classified as affected by bacterial pneumonia only based on the CRP values being higher than 100 μg/mL and respiratory symptoms; this would a severe conceptual mistake; as results from different studies and laboratories cannot be compared to each other, or because the criteria to name a disease are always based on the clinical presentation, diagnostic imaging, and in the case of septic conditions also on culture and of course also on laboratory results; these, however, with rare exceptions, may provide ancillary information and do not work alone as a diagnostic criterion. In addition, dogs with other airway diseases such as upper respiratory diseases may not experience a rise in CRP at all (e.g., rhinitis) [9].

In parvoviral enteritis, serum CRP concentration was associated with the prognosis in puppies [3]. During this study, CRP levels at admission and 12 and 24 h later were positively associated with the odds ratio and negatively associated with the survival time. Regarding differences between survivors and non-survivors, sensitivity and specificity of CRP concentration at 24 h after admission were 86.7% and 78.7%, respectively.

In conclusion, an increase in CRP concentration may only suggest the ongoing infection and encourage the clinician to order a culture and an antibiogram. Further diagnostic techniques such as susceptibility testing and targeting of pathogen(s) should be performed as often as possible because of the increasing global problem with antibiotic resistance.

## 6. Parasitic Etiology Diseases

The dynamics of CRP changes in many parasitic diseases have been extensively investigated. Especially *Babesia* invasions were explored, where it has been used to study the pathogenicity of the disease, to understand its diagnostic or prognostic role, or to assess responses to treatments and the severity of the disease. In 50 dogs with *Babesia canis*, the highest CRP concentration noted prior to imidocarb treatment exceeded 200 μg/mL [16].

In cases of leishmaniasis, no correlation seems to exist between CRP concentrations and antibody levels, but symptomatic dogs show higher CRP than the asymptomatic [14]. The finding that canine leishmaniasis doesn’t always result in changes in WBC is very important in a clinical setting, where CRP could be the only parameter suggesting an inflammation caused by the parasite [48]. However, when it comes to the treatment of this disorder, the CRP changes should be interpreted with caution [71]. Similarly, *Neospora caninum* invasion could influence APR leading to an increase in the concentration of APPs [12]. Some alteration was noted after *Toxocara canis* invasion [72], as well as in canine monocytic ehrlichiosis [73]. CRP is also observed to rise significantly in demodicosis [10]. Interestingly, in *Trypanosoma brucei* infections, CRP rose very slowly and did not peak until days 7–10 (>160 μg/mL) [15].

CRP has been researched in dogs with filariasis to differentiate those infected by different *Filarioidea* species, to differentiate dogs with latent infection from dogs with overt disease, dogs with complications (e.g., vena cava syndrome, thromboembolism, pulmonary hypertension) from dogs without complications. Several species of filarial worms such as *Dirofilaria immitis* may induce the 1–4-fold increase of CRP in dogs (69.9 μg/mL) whereas others do not, e.g., *Brugia pahangi* (12.9 μg/mL) [17].

In a dog suffering from *Anaplasma phagocytophilum* infection, the CRP rise was correlated with upcoming periods of immune-mediated polyarthritis (IMPA) symptoms. CRP values were also negatively correlated with corticosteroid treatment doses. CRP dropped 3-fold during the initial high dose 30-day prednisolone treatment (1–2 mg/kg/day). CRP rose again when the prednisolone dose was lowered to 0.5 mg/kg/day, and shortly the next symptomatic period occurred [74]; this leads to the conclusion that CRP could be used to predict the symptomatic periods in such diseases and also as a guide when establishing the lowest effective dose.

## 7. Surgery

Regardless of whether the injury of the tissue was caused by a surgical procedure or by accident, CRP can be expected to increase within 24 h as a result of the event alone [75,76]. CRP may be used for early detection of post-surgical complications, disregarding the type, length, or site of the surgery, before their clinical manifestation [77]. Regarding routine, less invasive procedures, the ovariohysterectomy (OH) in bitches did not influence the CRP level at all [78]. In another study, OH performed by laparoscopy induced a smaller CRP response than by laparotomy (2-fold) and the overall response was very mild. In both groups, CRP concentration started dropping after the 24 h. In the same study, dogs who underwent vasectomy showed barely any CRP response [79]. In beagles undergoing orchidectomy and treated with amoxicillin and carprofen, a 17-fold increase in the first 24 h post-surgery was noted (reaching over 100 μg/mL) [80]. The degree of severity of trauma does not always match the CRP level, but research proves that when the surgery is performed by an inexperienced surgeon, then the prolonged duration is the most likely reason for such inconsistency [81,82].

Various anesthetic protocols do not seem to affect post-surgical CRP levels [82,83]. In addition, single CRP measurements are of limited value regarding post-surgical inflammation and prognosis [84]. We believe that this claim can be extended beyond surgical procedures.

## 8. Orthopedic Diseases

In cases of lameness, CRP evaluation can facilitate differential diagnosis between immune-mediated or septic polyarthritis and other conditions that do not produce changes in CRP, such as intravertebral disc protrusion [9] as well paraplegia secondary to acute intervertebral disc extrusion [85]. Patellar luxation often requires surgery but does not cause severe trauma or inflammation by itself, as otherwise healthy dogs expecting surgery showed physiological CRP concentrations. Twenty-four hours after the surgery a 2–6-fold rise (median 92 μg/mL) was noted [86]. CRP may be a nonspecific biomarker for discospondylitis—but probably more sensitive than fever, leukocytosis, neutrophilia, and hyperglobulinemia. However, there is no association with the positive bacterial culture [87]. In 14/16 cases in dogs with bacterial discospondylitis the level of CRP was elevated (100.7 μg/mL), in contrast to 12 dogs with pyrexia and 6 experiencing leukocytosis [87]. Thus, it is still unclear if CRP can be used to differentiate between septic and non-septic articular diseases, or to guide clinical management (e.g., the necessity of surgery, after which it is also expected to rise).

## 9. Autoimmune Diseases

CRP increases in symptomatic (i.e., associated with SIRS) immune-mediated conditions, independent of the type of disease (e.g., immune-mediated hemolytic anemia—IMHA, immune-mediated thrombocytopenia—IMTP). Acute onset of IMHA is a life-threatening event. Evidence suggests it is accompanied by SIRS [88], which is supported by the high CRP findings in these dogs [9,20]. In most cases, dogs with IMHA display high values of CRP (143 ± 89 μg/mL) on the day of admission, which normalizes with treatment [11]. There is no significant difference in CRP levels in dogs with IMHA, IMTP, and immune-mediated polyarthritis (IMPA) [20]. In addition, CRP concentrations are almost 1-fold lower in dogs with IMHA receiving corticosteroids, but no correlation was found between initial CRP levels and survival until discharge.

CRP increases in Steroid Responsive Meningitis-Arteritis (SRMA) but not in meningoencephalitides of unknown origin, although the magnitude of increase in SRMA is similar to that of other inflammatory diseases. In a group of autoimmune diseases of the brain, meningoencephalitides of unknown origin, CRP often stays within reference values as systemic inflammation does not take place [89] in opposite to steroid-responsive meningitis arthritis (SRMA), where CRP rise is observed [21,90,91]. It is a valuable finding since the differential diagnosis between these two often requires a costly MRI scan, but SRMA-caused blood CRP shift is indistinguishable from one caused by systemic inflammatory disease [22].

CRP might be useful to differentiate between pemphigus foliaceous and superficial pyoderma, but only in more severe cases [92]. However, it is not a useful prognostic or treatment efficacy biomarker for canine atopic dermatitis [32]. Thus, the common conclusion of several studies is that CRP, in addition, alone or in addition to other diagnostic tests, may theoretically help differentiate between some potential diagnoses, regardless of disease types, the severity of clinical conditions, and the presence of complications (e.g., overwhelming bacterial dermatitis). CRP’s role in autoimmune diseases protection and prevention is also being investigated [93].

## 10. Neoplasia

The increase of CRP in patients with tumors (both in humans and in veterinary medicine) does not depend on the direct action of the tumor. Rather, CRP increases as a consequence of the secondary inflammatory/immune-mediated stimuli. In support of this hypothesis, it is widely demonstrated by dozens of studies (especially in humans) that increases in CRP are associated with increased staging, metastatization, or paraneoplastic complications (e.g., ulcers, immune depression, and so on). A study on a small population concluded that very often, there is no influence of the neoplastic condition on CRP concentration [65]. However, an extensive research paper published in 2020 shows that CRP concentrations in patients with immune-mediated and neoplastic diseases rise most frequently of all and to the greatest extent [9]. Hemangiosarcoma, nasal adenocarcinoma, cholangiocellular carcinoma, acute lymphoblastic leukemia, malignant histiocytosis, lymphoma, malignant mesothelioma, and intestinal adenocarcinoma were found to significantly increase CRP concentration. Of these cases, hematopoietic tumors might provoke the greatest elevation. In addition, disseminated tumors tend to cause higher spikes of CRP than localized tumors, which may not raise CRP at all (e.g., leiomyosarcoma, mammary gland tumors) [9,94]. In dogs with multicentric lymphoma, low, physiological CRP level was shown to be related to obtaining remission [33]. Furthermore, a correlation between high CRP and malignancy was suggested. In stage I, II, III, and IV of mammary tumors in bitches, a significant rise (approx. ten-fold) was observed only in stage IV. It was hypothesized that unless there is a metastasis or the tumor is greater than 5 cm in diameter, the lesions might be too poor of a stimulus for APPs production. It is unknown why stage IV showed higher concentrations than V [94].

However, the change in CRP concentration should only be an ancillary tool to predict the outcome, the response to treatment and the invasiveness of the tumor as well as to monitor over time the response to treatments and it cannot replace diagnostic imaging.

## 11. Other Diseases

Generally, in inflammatory bowel disease (IBD), CRP rises periodically along with symptoms and drops after successful prednisolone and metronidazole treatment, regardless of the organ affected [95]. No differences in CRP concentrations were found in dogs with IBD and other chronic gastrointestinal diseases among 51 dogs [23]. However, in a very recent study, the clinical value of CRP as indicative of duodenal histopathologic severity marker was evaluated and a positive correlation with canine IBD assessment index (CIBDAI) was confirmed [24].

Canine acute pancreatitis is a common disease in veterinary practice; however, the clinical presentations vary from subclinical to severe and life-threatening. Thus, there are differences between studies, and they may be due to the patient selection and inclusion criteria (e.g., different types/origins of pancreatitis, different phase, or duration of the disease and so on) and ultimately to the presence or absence (and severity) of SIRS. In 16 dogs with spontaneous acute pancreatitis, CRP measured on the day of admission was 2–5-fold higher and did not return to RI on day 5 [25]. Other authors report CRP within RI in dogs with pancreatitis, possibly due to a longer, subclinical period before the presentation [96]. In a recent study, it was suggested that serum canine pancreatic lipase immunoreactivity (cPLI) which is the most commonly used marker for pancreatitis confirmation, together with CRP could be used as objective biomarkers for clinical improvement in hospitalized dogs (8). In addition, a meta-analysis of 31 canine, rodent, and human studies provides evidence indicating the beneficial effect of corticosteroids on circulating CRP levels and hospitalization, whose administration during a pancreatitis episode is sometimes controversial [97].

CRP was found to be higher in dogs displaying pyrexia, regardless of the underlying cause (median 140 μg/mL) in 825 dogs, but with no linear correlation. Similarly, higher WBC count (>17,500) was correlated with higher CRP (385 dogs, median 38 μg/mL) and with no linear correlation as well. CRP was higher in dogs with low plasma albumin concentrations (albumin < 26 μg/mL, 128 dogs with median 50 μg/mL) than those with the higher value (408 dogs, median 8 μg/mL) [9]. Thus, CRP correlates with positive (fever) and negative (albuminemia) indicators of inflammation.

CRP was also investigated as a potential marker that would allow differentiating pyometra from cystic endometrial hyperplasia (CEH) [6]. Higher CRP values were expected to be reached in those with pyometra, as the underlying cause is septic, opposite to CEH. CEH is also not accompanied by systemic inflammation that often develops in pyometra. Indeed, the mean values were 3–4-fold higher for pyometra (200.28 ± 93.51 μg/mL and 53.51 ± 66.24 μg/mL for CEH). However, the values overlap.

Acute abdomen syndrome (AAS) is a group of nonspecific symptoms that may be caused by many diseases. In a study consisting of 32 dogs admitted to a clinic with AAS [96], 21 showed elevated CRP upon presentation. No correlation was found between CRP level and the later made diagnosis (e.g., pyometra, acute pancreatitis, gastric ulceration, etc.). None of the dogs whose CRP was within RI died. One dog with splenic hemangiosarcoma died (CRP 16.6 μg/mL) and among 6 euthanized dogs CRP ranged from 25 μg/mL to 202.5 μg/mL. In all dogs who survived until the second assay in 48–72 h but were euthanized/died later, CRP dropped from initial values. CRP also dropped to the greatest extent in dogs who died, rather than in survivors. In dogs with gastric dilatation, gastric volvulus and urolithiasis CRP was within the normal range, but other authors report a significant increase in CRP concentration 24 and 48 h after the gastric dilatation-volvulus surgery [98]. Thus, it may be concluded that CRP increases with the severity of the condition, although the rapid decrease over a short time may carry a negative prognostic value and should be taken into consideration by clinicians as well as researchers.

## 12. Conclusions

One of the most important conclusions drawn from our review is that canine CRP has a short half-life time and rises very shortly after the initial inflammatory factor affects homeostasis, which is consistent with current knowledge [2,16]. Generally, high CRP concentrations do not allow a conclusion of the underlying etiology or identification of bacterial inflammation [65]. Changes in CRP concentration in most of the reviewed studies have been summarized in Table 1.

CRP is widely used in veterinary medicine and increases in dogs when the disease is severe and associated with SIRS, independent of the type (primary inflammation or inflammation associated with other diseases). However, retrospective studies, even when big populations are evaluated, might have a limited value as the patients were not necessarily in the same stage of disease at the time of presentation, but were presented when the owner found it necessary—especially when neoplasia was involved. Regarding infectious diseases, it can seldom be assumed that the animal was naïve to the pathogen prior to the ongoing infection.

To obtain the full picture of the CRP dynamics, it would probably be necessary to perform future research in a controlled environment, where the studied condition would be artificially induced, and CRP be measured in specified time intervals. An ethical question would have to be answered if the potential benefits of such research outweigh the animal suffering caused by the condition. Based on our review, the answer is probably negative, as CRP is never crucial to obtain the diagnosis.

Some of the research suggests that it is not the CRP’s absolute value, but the decrease in time that might be a good prognostic factor, especially in life-threatening events, such as sepsis [68]. However, an unusually fast decrease might be a negative prognostic marker, since in dogs with acute abdomen those who experienced it died [96]. Nonetheless, the dynamics of CRP values, based on multiple measurements until recovery, are probably of much higher value as a guiding factor rather than a single measurement, often performed at admission and not repeated, when it is treated as an additional and less valuable diagnostic marker. Additionally, monitoring the concentration of CRP over time during the follow-up may allow differentiating responsive from non-responsive dogs, with the aforementioned exception.

Furthermore, we suggest that CRP cannot be evaluated as an isolated parameter or be the only diagnostic assay performed, and it should be complementary to good diagnostic practice and not a cheaper alternative to such. More extensive APPs profiles and multiple tests through the treatment should be recommended for the detection of inflammatory diseases.

## Figures and Tables

**Table 1:** CRP concentration values have been recorded in various studies.

Disease/Pathology	Noted CRP Level
Bordetella bronchoseptica infection	720 μg/mL [13] 20 μg/mL [10]
Aspiration bronchopneumonia	Symptomatic 65.03 μg/mL [10]
Leishmaniosis (mean)	Asymptomatic 30.08 μg/mL [14]
Trypanosoma brucei	>160 μg/mL [15]
Babesia canis	>200 μg/mL [16]
Dirofilaria immitis	69.9 μg/mL [17]
Parvovirus enteritis	Survivors—100.6 μg/mL Non-survivors—146.3 μg/mL [3]
Discospondylitis	100.7 μg/mL [18]
Immune-mediated hemolytic anemia-related systemic inflammatory response syndrome	Up to 435.1 μg/mL on day of admission [19]
Immune-mediated hemolytic anemia	11.70 ± 48.18 μg/mL [20]
Immune-mediated thrombocytopenia	11.55 ± 26.55 [20]
Immune-mediated polyarthritis	1.90 ± 7.00 μg/mL [20]
Steroid-responsive meningitis arthritis	85–327.1 μg/mL [19,20,21,22]
Inflammatory bowel disease	13.6 ± 7.6 μg/mL [23] 1.53–67.69 μg/mL [24]
Dietary responsive diarrhea	11.5 ± 3.9 μg/mL [23]
Antibiotic responsive diarrhea	13.8 ± 1.7 μg/mL [23]
Spontaneous acute pancreatitis	56.1 ± 12.7 ug/mL [25]
Pyometra	200.28 ± 93.51 μg [6]
Cystic endometrial hyperplasia	53.51 ± 66.24 μg/mL [6]

**Table 2:** A comparison of CRP assays validated in veterinary medicine. Abbreviations: POCT—Point of Care Testing.

Type of Test	Validation	Random-Access Availability	Duration	Prozone Effect	Lipemia and Hemolysis Interference	Inter-Assay Reliability (CV)
ELISA	[27,28]	−	Longer than acceptable for POCT	+	+ [2]	7.5–29% [27]
Immuno-turbidimetric	Canine-specific [29,30] POCT [27,28,31]	+	Short time	+	− [32]	<5% [32,33] <10% for all POCT, all 3 POCT correlated with the results from the ELISA reference method (0.97) but still did not achieve an acceptable coefficient [28,31]
	Human-specific validated for use in canines [34,35]	+	Short time	−	Not stated	Every batch of reagents has to be calibrated for valid results; CV <10% [35]
Colloidal gold immunochromatography	Species-specific rabbit anti-dog-CRP antibodies [36]	+	Short time	−	No interference in clinically relevant ranges of up to 800 mg/L bilirubin, 4 g/L hemoglobin and 8 g/L soybean oil (Intralipid) [36]	moderate to high CRP concentrations, ≤11%; ≤28% at low concentrations [36]
Capillary reversed passive latex agglutination test	[37]	−	Longer than acceptable for POCT	−	Not stated	10.28–12.40% [38]

+ means present; − means absent; CV: coefficient of variation; Point-of-care tests: POCT.

## Data Availability

Not applicable.
